# Comparison of Perioperative, Oncologic, and Functional Outcomes Following Robotic and Laparoscopic Intersphincteric Resection for Low Rectal Cancer: A Systematic Review and Meta-Analysis

**DOI:** 10.3390/jcm15166483

**Published:** 2026-08-21

**Authors:** Konstantinos Kossenas, Maximos Frountzas, Athanasios Syllaios, Nikolaos Pararas, Panagiotis Kokoropoulos, Dimosthenis Michelakis, Konstantinos Tsimogiannis, Dimitrios Symeonidis, Dimitrios Schizas

**Affiliations:** 1 Second Propedeutic Department of Surgery, Laiko General Hospital, National Kapodistrian University of Athens, 11527 Athens, Greece; 2 Department of Colorectal Surgery, Royal Marsden Hospital, NHS Foundation Trust, London SW3 6JJ, UK; 3 Colorectal Department, Addenbrooke’s Hospital, Cambridge University Hospitals NHS Trust, Cambridge CB2 0QQ, UK; 4 Third Department of Surgery, Attikon University Hospital, National and Kapodistrian University of Athens, 12462 Athens, Greece; 5 Fourth Department of Surgery, Attikon University Hospital, National and Kapodistrian University of Athens, 12462 Athens, Greece; 6 Department of Surgical Oncology, University General Hospital of Heraklion, University of Crete, 71500 Heraklion, Greece; 7 Department of Colorectal Surgery, St Luke’s Hospital, 55236 Thessaloniki, Greece; 8 Department of Surgery, University Hospital of Larissa, 41110 Larissa, Greece; 9 First Department of Surgery, National and Kapodistrian University of Athens, Laikon General Hospital, 11527 Athens, Greece; schizasad@gmail.com

**Keywords:** robotic surgery, sphincter preserving, rectal cancer, laparoscopic surgery, intersphincteric, functional outcomes, quality of life, oncological outcomes, Wexner

## Abstract

**Background**: Intersphincteric resection (ISR) is a technically demanding sphincter-preserving procedure for low rectal cancer. While robotic surgery may offer technical advantages, evidence comparing robotic ISR (R-ISR) and laparoscopic ISR (L-ISR) remains limited. This study aimed to compare perioperative, oncologic, and functional outcomes between R-ISR and L-ISR. **Methods**: A systematic review and meta-analysis was conducted in accordance with PRISMA 2020. PubMed, Scopus, and Cochrane Library were searched up to 1 March 2026. Comparative studies evaluating R-ISR versus L-ISR in adult patients with low rectal cancer were included. Random-effects models were used to calculate mean differences (MDs) and odds ratios (ORs) with 95% confidence intervals (CIs). Heterogeneity was assessed using I^2^. Subgroup and sensitivity analyses were performed. **Results**: Seven studies were included. R-ISR was associated with a significantly longer operative duration (MD 34.53 min, 95% CI 7.48 to 61.59; *p* = 0.02; I^2^ = 81%), a lower rate of overall complications (OR 0.78, 95% CI 0.61 to 0.99; *p* = 0.04; I^2^ = 0%), a statistically lower Wexner score at 12 months, although the magnitude of the difference was small and its clinical significance uncertain (MD −1.53, 95% CI −2.54 to −0.51; *p* = 0.02; I^2^ = 0%), and a slightly lower lymph node yield (MD −1.06, 95% CI −2.05 to −0.08; *p* = 0.04; I^2^ = 65%). No significant differences were observed in blood loss (MD −8.15, 95% CI −23.03 to 6.73; *p* = 0.20; I^2^ = 37%), conversion to open surgery (OR 0.35, 95% CI 0.02 to 6.02; *p* = 0.13; I^2^ = 0%), anastomotic leakage (OR 0.92, 95% CI 0.61 to 1.37; *p* = 0.60; I^2^ = 0%), length of hospital stay (MD −0.48, 95% CI −1.16 to 0.21; *p* = 0.12; I^2^ = 0%), and CRM positivity (OR 0.93, 95% CI 0.01 to 69.04; *p* = 0.87; I^2^ = 0%). Subgroup analyses in experienced surgeons and high-volume centers demonstrated no statistically significant differences across outcomes. Sensitivity analyses showed that several results were not robust. **Conclusions**: Evidence to date does not show superiority of robotic or laparoscopic ISR. Robotic ISR was associated with longer operative time and lower overall complication rates but most perioperative and oncologic outcomes were comparable. After robotic ISR, the 12-month Wexner scores were statistically lower, although the magnitude of this difference was small and the clinical significance uncertain. These results should be interpreted with caution given the limited non-randomized evidence base. PROSPERO Registration: CRD420261359130.

## 1. Introduction

Rectal cancer is the third most commonly diagnosed cancer worldwide [1] and tumors located in the distal rectum can be challenging to control surgically due to their proximity to the anal sphincter complex and surrounding structures in the pelvis [2]. Historically these tumors have been managed by an abdominoperineal resection (APR), where patients require a permanent colostomy leading to a poor quality of life [3]. However, with advancing surgical techniques and a growing emphasis on sphincter preservation and improved patient quality of life, the traditional use of the APR for the management of low rectal cancer is increasingly being questioned. The technique of intersphincteric resection (ISR) has evolved for the management of a select group of patients with distal rectal cancer, allowing for an oncologically safe resection and the preservation of continence [4,5].

Although the potential benefits of an ISR approach are clear, the procedure is technically demanding [6]. For patients with rectal cancer requiring surgery, the surgeon must perform precise dissection within a narrow pelvic field, clearly identify the intersphincteric plane, and protect critical autonomic nerves to avoid urinary and anorectal dysfunction that may be permanent [7].

The minimally invasive surgical techniques for rectal cancer have been widely adopted and the number of reports on laparoscopic rectal surgery has increased in recent years [8]. Laparoscopic rectal surgery provides several advantages over open surgery, including less postoperative pain and quicker recovery [9]. However, conventional laparoscopy has several limitations including two-dimensional visual fields, restriction of instrument movement, and uncomfortable surgical positions [10]. These limitations can make it difficult to carry out precise dissection within the deep pelvis, particularly during circumferential resection in the deep pelvis with nerve-sparing, which requires a high degree of technical precision [11].

Recent technological advancements in robotic surgical systems may overcome several limitations of conventional laparoscopy in performing ISR. The robotic system offers surgeons high-definition 3D visualization, wristed instrumentation, surgical tremor filtering, and improved ergonomics [12,13,14,15,16]. This may allow for more precise surgical dissection in the deep pelvic space and better postoperative function [17,18].

Several studies comparing robotic and laparoscopic surgery for rectal cancer have been published. However, the majority of those studies focused on total mesorectal excision (TME), along with predictive models, and not on individual segments such as intersphincteric resection (ISR) [19,20,21]. Despite the increasing adoption of robotic surgery, a significant gap persists in the literature regarding the comparative effectiveness of robotic versus laparoscopic intersphincteric resection (ISR), particularly with respect to functional outcomes. This systematic review and meta-analysis therefore aimed to compare robotic and laparoscopic ISR for low rectal cancer, focusing on perioperative, oncologic, and functional outcomes, in order to address this gap in the literature. Given the technical complexity of ISR, this study provides a comprehensive evaluation of the potential advantages and limitations of robotic surgery in this demanding surgical setting.

## 2. Materials and Methods

### 2.1. Study Design

This systematic review and meta-analysis was conducted in accordance with the PRISMA 2020 (Preferred Reporting Items for Systematic Reviews and Meta-Analyses) statement and the Cochrane Handbook [22,23]. The study was prospectively registered with PROSPERO (registration: CRD420261359130).

### 2.2. Literature Search

A comprehensive literature search was performed in PubMed, Scopus and the Cochrane Library up to the 1 March 2026, using a combination of keywords and relevant MeSH terms. Search strategies are presented in detail in the Supplementary Table S2. Additionally, two independent authors manually searched the reference lists of included studies for relevant additional publications.

### 2.3. Eligibility Criteria

We performed a search for clinical studies that compared robotic intersphincteric resection (R-ISR) with laparoscopic intersphincteric resection (L-ISR) in patients with low rectal cancer. Inclusion criteria were: (1) clinical studies comparing R-ISR with L-ISR surgery; (2) studies including adult patients (≥18 years) with low rectal cancer; (3) studies in which all patients underwent intended curative surgical treatment with intersphincteric resection; (4) studies that were either randomized controlled trials (RCTs), prospective or retrospective comparative cohort studies; and (5) studies in which at least one of the indicated outcomes were reported (perioperative, oncologic, functional). Primary outcomes for this study were considered the LARS and Wexner score at 6 and 12 months respectively, number of harvested lymph nodes and overall complications. Exclusion criteria were: (1) non-comparative studies (including single-arm studies), (2) studies that were not ISR (i.e., abdominoperineal resection, low anterior resection), (3) studies in which ISR data were not available. Studies comparing robotic or laparoscopic to open surgery were also excluded. Additionally, studies focusing on benign colorectal diseases and malignancies other than colorectal cancer were not included. Review articles, commentaries, editorials, conference abstracts, letters to the editor and case reports, animal experiments or studies published in languages other than English were also excluded. Duplicated data (published in different articles or same authors) were excluded, except for the most recent publication.

### 2.4. Study Selection, Data Extraction and Outcomes

Titles and abstracts were screened by two independent reviewers. The full-text articles were then screened by the same two reviewers and were marked for inclusion if they met the inclusion criteria. Data were extracted in predefined extraction forms which included study characteristics (author/year/study design/center type) and patient demographics (study sample/age/sex distribution/BMI/tumor height/neoadjuvant chemoradiotherapy status). Surgical characteristics extracted included surgeon experience and the robotic surgery approach (total/robot-assisted) as well as center volume where reported. Outcomes of interest included perioperative outcomes, oncologic outcomes and functional outcomes. Preference was given to data from cohorts that had been propensity score-matched or subjected to inverse probability of treatment weighting where available to reduce confounding and allow for more valid comparison between groups.

### 2.5. Risk of Bias Assessment and Statistical Analysis

The studies included in this review were assessed using the ROBINS-I checklist [24]. For each included study, risk of bias was assessed across seven domains and an overall risk of bias judgment was assigned. Bias was assessed independently by two reviewers and any disagreements were resolved by discussion. Pooled analyses were performed using Review Manager Web software (5.4) [25]. Dichotomous data are presented as odds ratios (ORs) with 95% confidence intervals (CIs) and continuous data as mean difference (MD) with 95% CI. A random-effects model was used for the meta-analyses to account for between-study variability. The I^2^ was calculated to evaluate the between-study heterogeneity. An I^2^ value > 60% indicated significant between-study heterogeneity. Two pre-specified subgroup analyses were conducted based on surgeon experience and center volume. Additionally, two sensitivity analyses were conducted by (1) excluding studies with high risk of bias and (2) “leave one out” approach. Funnel plots were used to assess publication bias only in the primary cohort. Egger’s test was not conducted due to the small number of studies identified (n < 10). Finally, where necessary, means and standard deviations were estimated using the methods of Wan et al. [26].

## 3. Results

### 3.1. Search Results

**Figure 1:**
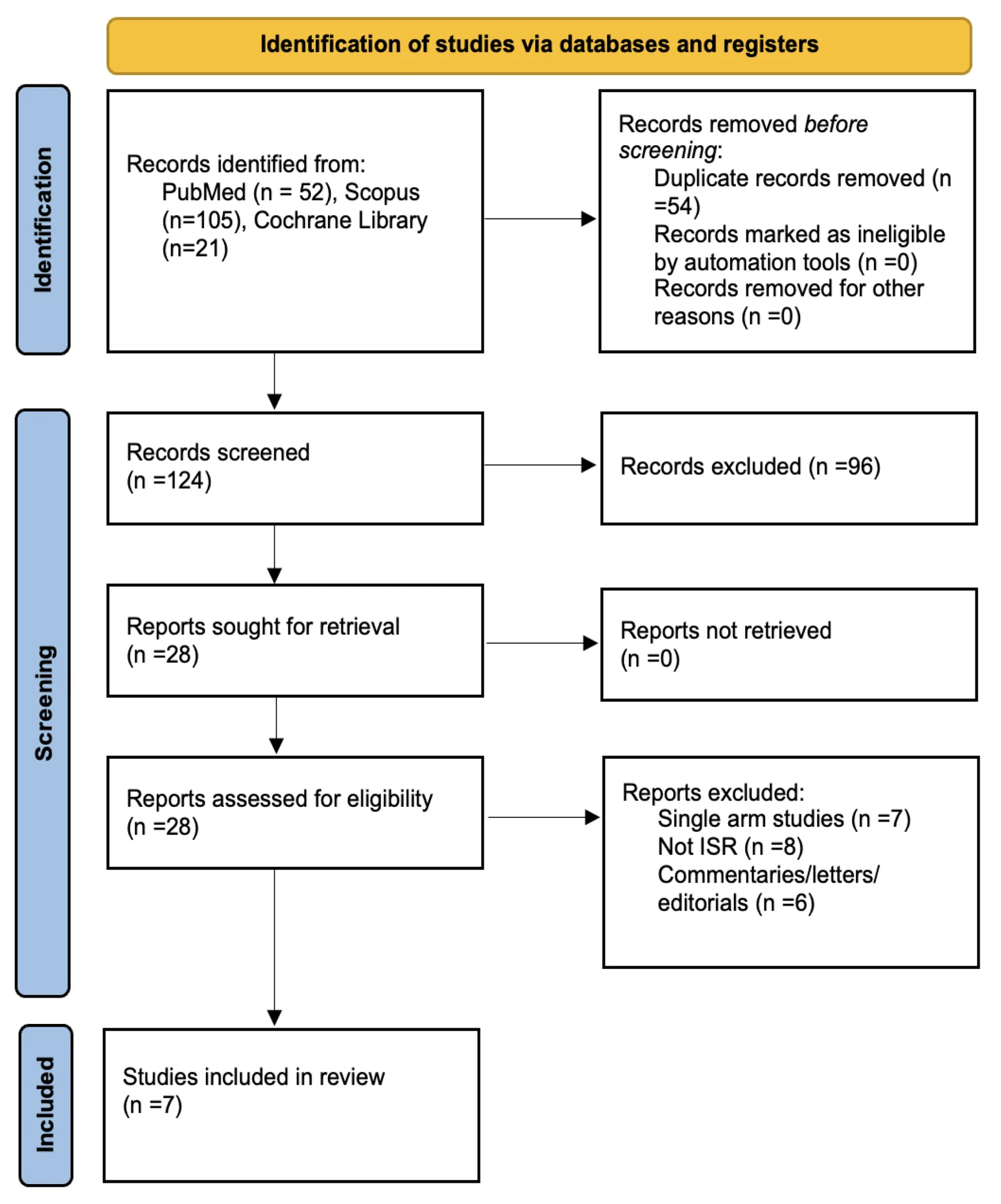
PRISMA Flowchart 2020.

A total of 178 records were identified through database searching, with 54 duplicates removed, leaving 124 studies for screening. After screening, 28 reports were assessed for eligibility, of which 21 were excluded for predefined reasons. Ultimately, 7 studies met the inclusion criteria and were included in the final review (Figure 1).

### 3.2. Baseline Characteristics

Seven studies were included in this review [27,28,29,30,31,32,33]. The majority were retrospective cohort studies (n = 5), with one propensity score-matched study and one inverse probability of treatment weighting analysis. The majority of studies were single-center, with only one multicenter study identified. Sample sizes were generally similar in the robotic and laparoscopic groups across the studies. Baseline patient characteristics were well balanced within individual studies, with mean age from 46 to 63 years and a predominance of male patients. In each study the groups were similar in terms of body mass index and tumor height. The use of neoadjuvant chemoradiotherapy varied greatly across studies. Variability was also observed with regard to the surgeon’s experience. Several studies were carried out in high-volume centers but this information was not consistently reported. Overall, the included studies showed acceptable baseline comparability, although heterogeneity in neoadjuvant treatment and surgeon’s experience should be taken into account when interpreting the pooled results (Table 1).

### 3.3. Meta-Analysis

Overall, robotic ISR was associated with a significantly longer operative duration compared to laparoscopic ISR (MD 34.53 min, 95% CI 7.48 to 61.59; *p* = 0.02), with substantial heterogeneity (I^2^ = 81%) and evidence of funnel plot asymmetry. No significant differences were observed in blood loss, conversion to open surgery, anastomotic leakage, or length of hospital stay. Robotic ISR demonstrated a lower rate of overall complications (OR 0.78, 95% CI 0.61 to 0.99; *p* = 0.04) and a modest reduction in Wexner score at 12 months (MD −1.53, 95% CI −2.54 to −0.51; *p* = 0.02), although functional outcomes were based on a limited number of studies. Lymph node yield was slightly lower in the robotic group (MD −1.06, 95% CI −2.05 to −0.08; *p* = 0.04) with moderate heterogeneity (I^2^ = 65%). Funnel plot assessment suggested symmetry for most outcomes, with mild asymmetry observed for anastomotic leakage and lymph node harvest, while several outcomes were not assessable due to the small number of studies. Subgroup analyses in experienced surgeons and high-volume centers showed consistent trends without statistically significant differences, although these analyses were limited by small sample sizes and substantial heterogeneity (Table 2). Funnel plots for publication bias can be found in the Supplementary Materials.

### 3.4. Primary Outcomes

**Figure 2:**
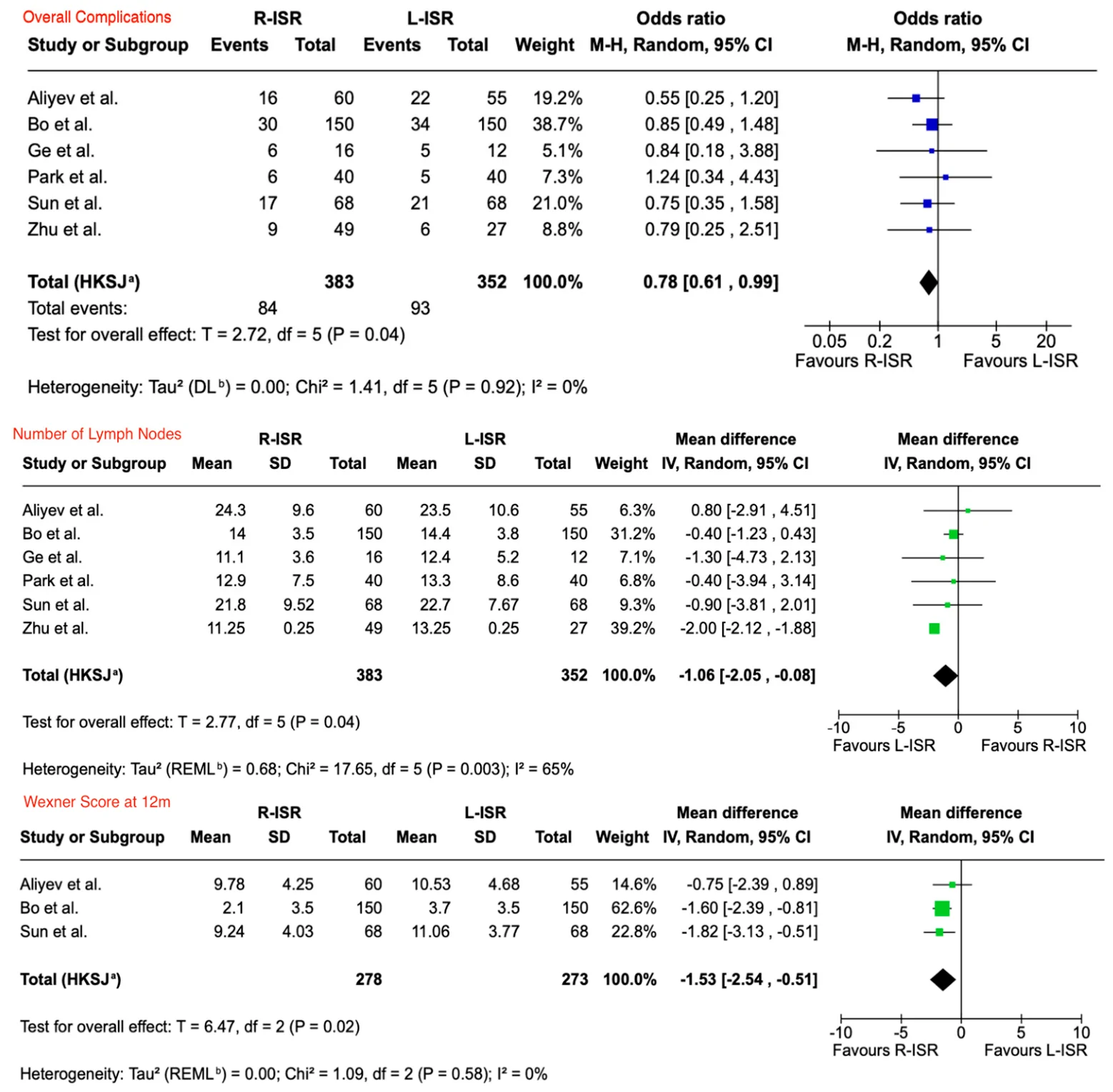
Forest plots of primary outcomes [27,28,29,30,31,33]. Green squares; continuous variables, blue squares; dichotomous variables. ^a^ Hartung–Knapp–Sidik–Jonkman method used for confidence intervals. ^b^ Between-study variance (τ^2^) estimated using restricted maximum likelihood.

Figure 2 displays the forest plots of the primary outcomes (overall complications, number of lymph nodes harvested and Wexner Score at 12 months). The Forest plots for the secondary outcomes can be found in the Supplementary Materials.

### 3.5. Risk of Bias Assessment

The risk of bias assessment using the ROBINS-I tool demonstrated that the overall methodological quality of the included studies ranged from moderate to serious. Three studies were judged to have an overall serious risk of bias, primarily driven by confounding and limitations in participant selection, while the remaining studies were assessed as having a moderate risk of bias. Across studies, the risk of bias due to confounding was the most prominent concern, reflecting the non-randomized design of the included cohorts. In contrast, the classification of interventions and deviations from intended interventions were consistently rated as low risk. Moderate concerns were noted in domains related to missing data, outcome measurement, and selective reporting. Overall, these findings highlight the inherent limitations of observational evidence and underscore the importance of interpreting the pooled results with caution (Table 3).

### 3.6. Sensitivity Analysis

A sensitivity analysis was performed for outcomes with substantial heterogeneity, and a secondary analysis was performed by excluding studies with serious risk of bias. Sensitivity analyses demonstrated that the results for operative duration were not robust, as exclusion of individual studies [31,33] rendered the effect non-significant while heterogeneity remained high. For blood loss, exclusion of Bo et al. [28] resulted in a significant effect favoring robotic ISR and eliminated heterogeneity (I^2^ = 0%). Lymph node harvest findings were sensitive to multiple studies; exclusion of Bo et al. [28] reduced heterogeneity substantially (I^2^ = 22%) while maintaining significance. Furthermore, exclusion of studies with serious risk of bias led to loss of statistical significance across all outcomes, with heterogeneity decreasing for blood loss and lymph node yield but increasing for operative duration (Table 4).

### 3.7. GRADE Assessment and Evidence Certainty

We used the GRADE framework [34] to rate the certainty of evidence, which ranged from moderate to very low across outcomes. The most common reasons for downgrading certainty were imprecision, inconsistency and potential publication bias. Overall complications were moderate-certainty evidence, and most other outcomes were low- or very-low certainty. Subgroup analyses restricted to experienced surgeons and high-volume centers did not substantially increase certainty, largely because of the small number of contributing studies, wide confidence intervals, and persistent heterogeneity (Table 5).

## 4. Discussion

The mean increase in operative time for R-ISR was 34 min, which was not robust following subgroup analysis. There were no significant differences between groups for blood loss (8 mL less with R-ISR), conversion to open surgery, anastomotic leak, or postoperative hospital stay. However, there was a significant decrease in overall complications with R-ISR (22% less odds of any complication), although this benefit was not consistently demonstrated in subgroup and sensitivity analyses. The number of lymph nodes retrieved was marginally lower in the R-ISR group by approximately one node compared to laparoscopy, a difference that is unlikely to be clinically meaningful or to compromise oncologic adequacy. At 12 months, Wexner score was statistically lower in R-ISR treated patients (pooled mean difference of −1.53 points; 95% CI −2.54 to −0.51). However, the difference was small in absolute terms and its clinical significance is unclear. Thus, this finding should not be interpreted as demonstrating a clinically meaningful improvement in continence after robotic ISR., compared to L-ISR. All included studies were non-randomized studies with moderate-to-serious risk of bias, and a number of statistically significant findings were not significant in sensitivity analyses. The observed associations should therefore be interpreted as hypothesis-generating rather than as evidence of superiority of either surgical approach.

Our results offer new and complementary perspectives to the current literature. In relation to Dimitrijevic et al., [35] who showed better preservation of the pelvic autonomic nerves and short-term urinary and sexual function with laparoscopic versus open rectal surgery, our study extends this concept in a purely minimally invasive context which may suggest robotic ISR may have an additional functional benefit over laparoscopy as measured by improved Wexner scores at 12 months. Our analysis is limited to intersphincteric resection, a highly specialized, sphincter-preserving procedure, and includes perioperative, oncologic, and longer-term functional outcomes, unlike their broader inclusion of multiple rectal procedures and short-term outcomes. Our study showed similar oncologic outcomes between robotic and laparoscopic ISR with a slight functional benefit with robotics. Liao et al. [36] reported better oncologic outcomes with TaTME but worse functional results (higher major LARS), indicating that the balance between oncologic and functional results may vary with the surgical approach. These differences probably reflect differences in patient selection and operative technique as TaTME is intended for mid-to-low rectal cancers by way of a transanal approach whereas ISR is a more technically demanding distal sphincter-preserving operation. Our findings also corroborate those of Madarasz et al. [37] who demonstrated longer operative time and lower complication rates in robotic surgery but comparable oncologic results; however, we did not find differences in hospital stay or conversion rates. Importantly, our study is different because we focus exclusively on ISR and provide a synthesis of multicenter comparative evidence, while Madarasz et al. [37] investigated a broader rectal cancer cohort in a single-center retrospective design. In contrast to previous meta-analyses by Zhang et al. [38] and Lee et al. [39], our study provides an updated synthesis with recent evidence up to 2026 and additional subgroup and sensitivity analyses. Zhang et al. [38] and Lee et al. [39] reported fewer blood losses and conversion rates in robotic ISR. Nevertheless, our analysis revealed no significant differences in these outcomes. Crucially, our study is the first to show a small improvement in functional outcomes (Wexner score at 12 months) with robotic ISR. Overall, current evidence does not demonstrate the superiority of either surgical platform for ISR. Robotic ISR was associated with a longer operative duration, but the reductions in overall complications and Wexner score were modest and not consistently sustained in subgroup and sensitivity analyses. Conversely, the approximate one-node difference in lymph node yield favoring laparoscopy is unlikely, in isolation, to represent a clinically important oncologic advantage. Therefore, these results should be viewed as indicating broadly similar short-term and oncologic results between the approaches, rather than as evidence in support of either robotic or laparoscopic ISR. Differences in the studied population and procedure may also explain the apparent discrepancy with studies reporting potential advantages of robotic rectal surgery. The majority of the broader literature evaluates robotic TME or rectal resection in general, while the present analysis was restricted to ISR alone, a technically unique operation that entails intersphincteric dissection and coloanal reconstruction. Therefore, evidence for benefits of robotics in larger populations of rectal surgery patients cannot necessarily be directly extrapolated to ISR.

### 4.1. Strengths and Limitations

The present study has several strengths. It offers a concise, current synthesis of the available data comparing robotic versus laparoscopic intersphincteric resection (ISR), an area of minimally invasive rectal surgery that remains relatively underexplored. By restricting inclusion to ISR, it increases clinical relevance and provides a procedure-specific assessment of perioperative, oncologic and functional outcomes. The use of propensity score-matched and inverse probability of treatment weighted cohorts enhanced comparability and predefined subgroup and sensitivity analyses enhanced methodological rigor. Another major strength is the inclusion of functional outcomes given their importance in sphincter-preserving surgery. But certain limitations must be recognized. All the studies included were non-randomized and mostly retrospective, resulting in moderate to very low certainty of the evidence and risk of bias. One study (Kazi et al.) did not report several outcomes of interest and functional outcomes were only measured at end of follow-up and not at standardized time points (e.g., 6 or 12 months), limiting comparability. In addition, some relevant functional outcomes such as major LARS at 6 and 12 months, Wexner score at 6 months, Kirwan score at 6 and 12 months, IPSS at 6 and 12 months, and IIEF score at 6 and 12 months were not meta-analyzed due to inconsistent reporting. Finally, the small number of studies for some outcomes and heterogeneity in surgeon experience, center volume, neoadjuvant therapy, and surgical technique further limit the robustness and generalizability of the findings. Lastly, the available data did not allow for a robust comparison of health-related quality of life between approaches, which is particularly important when considering a sphincter-preserving procedure where small differences in functional scores may not necessarily translate into patient-perceived benefit.

### 4.2. Future Directions and Proposal of a Prospective Study

As functional results are important in sphincter-preserving surgery, future studies should be prospective multicenter studies with standardized and complete assessment of functional outcomes. The primary end-point for assessment of bowel function was the LARS score and continence was also assessed with the Wexner score. Urinary and sexual function should be assessed with validated instruments like IPSS, IIEF-5 and FSFI. Quality-of-life measures include EORTC QLQ-C30 and QLQ-CR29. Outcomes should be reported as continuous values and clinically meaningful categories (e.g., major LARS) and evaluated at standardized time points including baseline, 6 months, 12 months and ideally 24 months after restoration of bowel continuity to evaluate functional recovery over time. Based on the observed difference of approximately 1.5 points in Wexner score, a future adequately powered study would need approximately 224 patients (112 per group), increasing to approximately 264 patients when accounting for potential loss to follow-up, to reliably detect clinically meaningful differences between robotic and laparoscopic ISR.

## 5. Conclusions

Robotic intersphincteric resection has a longer operative time but similar perioperative and oncologic outcomes compared with laparoscopic ISR. Evidence to date does not show superiority of robotic or laparoscopic ISR. Robotic ISR was associated with longer operative times and lower overall complication rates, with most perioperative and oncologic outcomes being similar. Robotic ISR showed statistically significant lower Wexner scores at 12 months, but the absolute difference was small and clinical significance is unknown. The findings should be interpreted with caution given the non-randomized nature of the evidence base, the moderate-to-serious risk of bias and the limited robustness of several of the pooled estimates. High-quality randomized or prospective multicenter studies with standardized functional and quality of life outcomes are needed to establish superiority.

## Figures and Tables

**Table 1:** Baseline characteristics of the included studies.

Author (Year)	Design	Center	Sample Size (R/L)	Age (R/L)	Male % (R/L)	BMI (R/L)	Tumor Height cm (R/L)	Neoadjuvant CRT % (R/L)	Experiences Surgeon (R/L) [Yes/No)	Total/Assisted Robotic	High-Volume Center
Zhu et al. (2025) [27]	RCS	SC	49/27	56.67/53.56	57.1/70.4	22.67/22.87	3.94/3.85	100/100	Yes/Yes	Robot-assisted	Yes
Yang Bo et al. (2025) [28]	RCS	SC	150/150	58.3 ± 10.4/60.2 ± 10.8	62.7%/64.0%	23.6 ± 3.2/23.3 ± 3.7	3.94 ± 0.48/5.66 ± 0.47	22.0/32.0	Yes/Yes	Robot-assisted ISR	Yes
Ge et al. (2024) [29]	RCS	SC	16/12	57.1 ± 15.2/58.0 ± 9.5	50.0%/58.3%	23.8 ± 2.8/24.5 ± 3.1	3.2 ± 1.0/2.5 ± 0.9	0/8.3	NR/NR	Robotic-assisted	NR
Sun et al. (2024) [30]	PSM	MC	68/68	62.5 ± 8.7/63.1 ± 8.0	70.6%/67.6%	23.97 ± 3.18/23.93 ± 2.12	2.9 ± 0.61/3.2 ± 0.69	0/0	Yes/Yes	Robotic-assisted	NR
Aliyev et al. (2022) [31]	RCS	SC	60/55	60.0 ± 10.2/58.0 ± 9.7	70.0/65.5	23.0 ± 2.8/24.0 ± 2.4	3.0 ± 0.6/3.5 ± 0.7	100/94.5	Yes/Yes (single expert colorectal surgeon)	Total robotic (da Vinci Si/Xi)	Yes
Kazi et al. (2023) [32]	IPTW	SC	130/132 (IPTW weighted)	46.42 ± 12.19/46.34 ± 12.55	73.5/74.1	23.69 ± 3.62/23.67 ± 3.36	3.3 ± 1.52/3.3 ± 1.49	84.6/84.9	No/Yes (not reported by group; robotic experience lower overall, ~4:1 lap:robot ratio)	Total robotic (da Vinci Xi)	Yes
Park et al. (2013) [33]	RCS	SC	40/40	57.3 ± 12.1/63.6 ± 10.6	70.0/62.5	23.9 ± 2.4/24.3 ± 3.1	3.4 ± 1.1/3.6 ± 1.3	80.0/50.0	Initial robotic experience included	Robotic-assisted	NR

RCS, retrospective cohort study; PSM, propensity score matching; IPTW, inverse probability of treatment weighting; SC, single center; MC, multicenter; R/L, robotic/laparoscopic; BMI, body mass index; CRT, chemoradiotherapy; NR, not reported.

**Table 2:** R-ISR vs. L-ISR.

Outcome	Number of Studies	MD/OR [95%CI]	*p* Value	I^2^	Funnel Plot Interpretation
Overall R-ISR vs. L-ISR					
Operative duration	5	34.53 [7.48, 61.59]	0.02	81	asymmetry
Blood loss	5	−8.15 [−23.03, 6.73]	0.2	37	symmetry
Conversion to open surgery	2	0.35 [0.02, 6.02]	0.13	0	inconclusive (2 studies only)
Overall complications	6	0.78 [0.61, 0.99]	0.04	0	symmetry
Anastomotic leakage	6	0.92 [0.61, 1.37]	0.6	0	mild asymmetry
Length of hospital stay	5	−0.48 [−1.16, 0.21]	0.12	0	symmetry
Lymph nodes harvested	6	−1.06 [−2.05, −0.08]	0.04	65	mild asymmetry
CRM +	2	0.93 [0.01, 69.04]	0.87	0	inconclusive (2 studies only)
Wexner 12 m	3	−1.53 [−2.54, −0.51]	0.02	0	inconclusive (3 studies only)
Experienced surgeons					
Operative duration	3	25.42 [−16.60, 67.44]	0.12	83	Not assessed
Blood loss	3	−8.09 [−35.24, 19.06]	0.33	55	
Conversion to open surgery	0	n/a			
Overall complications	4	0.74 [0.54, 1.02]	0.06	0	
Anastomotic leakage	4	0.84 [0.48, 1.47]	0.4	0	
Length of hospital stay	3	−0.36 [−0.95, 0.24]	0.12	0	
Lymph nodes harvested	4	−1.05 [−2.73, 0.63]	0.14	78	
CRM +	0	n/a			
Wexner 12 m	0	n/a			
High-volume centers					
Operative duration	2	28.29 [−185.17, 241.75]	0.34	88	Not assessed
Blood loss	2	1.23 [−75.47, 77.94]	0.87	0	
Conversion to open surgery	1	n/a			
Overall complications	3	0.74 [0.41, 1.35]	0.17	0	
Anastomotic leakage	3	0.83 [0.32, 2.15]	0.48	0	
Length of hospital stay	2	−0.59 [−1.53, 0.36]	0.08	0	
Lymph nodes harvested	3	−1.02 [−4.05, 2.02]	0.29	87	
CRM +	1	n/a			
Wexner 12 m	2	−1.44 [−5.67, 2.79]	0.14	0	

**Table 3:** Risk of bias assessment.

Study	Confounding	Selection of Participants	Classification of Interventions	Deviations from Intended Interventions	Missing Data	Measurement of Outcomes	Selection of Reported Results	Overall ROBINS-I
Zhu et al., 2025 (ISR subgroup) [27]	Serious	Moderate	Low	Low	Moderate	Moderate	Moderate	Serious
Yang Bo et al. (2025) [28]	Serious	Moderate	Low	Low	Moderate	Moderate	Moderate	Serious
Ge et al. (2024) [29]	Serious	Moderate	Low	Low	Low	Moderate	Moderate	Serious
Sun et al. (2024) [30]	Moderate	Moderate	Low	Low	Low	Moderate	Moderate	Moderate
Aliyev et al. (2022) [31]	Moderate	Moderate	Low	Low	Moderate	Moderate	Moderate	Moderate
Kazi et al. (2023) [32]	Moderate	Moderate	Low	Low	Moderate	Moderate	Moderate	Moderate
Park et al. (2013) [33]	Serious	Moderate	Low	Low	Low	Moderate	Moderate	Serious

**Table 4:** Sensitivity analyses.

Sensitivity Analysis “Leave One Out”	
Operative duration	Exclusion of Aliyev et al. [31] and Park et al. [33] rendered the pooled estimate non-significant, but the heterogeneity remained high.
Blood loss	Exclusion of Bo et al. [28] rendered the effect significant in favor of R-ISR and dropped heterogeneity to 0%.
Lymph nodes harvested	Exclusion of Bo et al. [28] dropped heterogeneity to 22% from 65%, with results remaining significant in favor of L-ISR, whereas exclusion of Ge et al., [29] Park et al., [33] and Sun et al., [30] rendered the pooled estimated non-significant whilst increasing heterogeneity. Exclusion of Zhu et al., [27] rendered the pooled estimated non-significant but dropped heterogeneity to 0%.
Sensitivity analysis: exclusion of studies with serious risk of bias	
operative duration	Exclusion of studies with high risk of bias rendered the pooled estimate non-significant and heterogeneity spiked to 87%.
blood loss	Exclusion of studies with high risk of bias rendered the pooled estimate non-significant and heterogeneity dropped to 0%.
lymph nodes harvested	Exclusion of studies with high risk of bias rendered the pooled estimate non-significant and heterogeneity dropped to 0%.

**Table 5:** GRADE assessment for overall cohort R-ISR vs. L-ISR.

Outcome	Studies	Effect (95% CI)	Inconsistency	Imprecision	Publication Bias	Certainty
Operative duration	5	34.53 (7.48, 61.59)	Very serious (I^2^ = 81%)	Serious	Suspected (asymmetry)	Very low
Blood loss	5	−8.15 (−23.03, 6.73)	Not serious	Serious	Not suspected (symmetry)	Low
Conversion to open	2	0.35 (0.02, 6.02)	Not serious	Very serious	Not assessable	Very low
Overall complications	6	0.78 (0.61, 0.99)	Not serious	Borderline	Not suspected (symmetry)	Moderate
Anastomotic leakage	6	0.92 (0.61, 1.37)	Not serious	Serious	Possible (mild asymmetry)	Low
Length of stay	5	−0.48 (−1.16, 0.21)	Not serious	Serious	Not suspected (symmetry)	Low
Lymph nodes harvested	6	−1.06 (−2.05, −0.08)	Serious (I^2^ = 65%)	Serious	Possible (mild asymmetry)	Low
CRM positive	2	0.93 (0.01, 69.04)	Not serious	Very serious	Not assessable	Very low
Wexner score (12 months)	3	−1.53 (−2.54, −0.51)	Not serious	Serious	Not assessable	Low
**Subgroup: Experienced surgeons cohort**						
Operative duration	3	25.42 (−16.60, 67.44)	Very serious (I^2^ = 83%)	Very serious	Not assessable	Very low
Blood loss	3	−8.09 (−35.24, 19.06)	Serious (I^2^ = 55%)	Very serious	Not assessable	Very low
Overall complications	4	0.74 (0.54, 1.02)	Not serious	Serious	Not assessable	Low
Anastomotic leakage	4	0.84 (0.48, 1.47)	Not serious	Serious	Not assessable	Low
Length of stay	3	−0.36 (−0.95, 0.24)	Not serious	Serious	Not assessable	Low
Lymph nodes harvested	4	−1.05 (−2.73, 0.63)	Very serious (I^2^ = 78%)	Serious	Not assessable	Very low
**Subgroup: High-Volume centers**						
Operative duration	2	28.29 (−185.17, 241.75)	Very serious (I^2^ = 88%)	Very serious	Not assessable	Very low
Blood loss	2	1.23 (−75.47, 77.94)	Not serious	Very serious	Not assessable	Very low
Overall complications	3	0.74 (0.41, 1.35)	Not serious	Serious	Not assessable	Low
Anastomotic leakage	3	0.83 (0.32, 2.15)	Not serious	Serious	Not assessable	Low
Length of stay	2	−0.59 (−1.53, 0.36)	Not serious	Serious	Not assessable	Low
Lymph nodes harvested	3	−1.02 (−4.05, 2.02)	Very serious (I^2^ = 87%)	Serious	Not assessable	Very low
Wexner score (12 months)	2	−1.44 (−5.67, 2.79)	Not serious	Very serious	Not assessable	Very low

## Data Availability

All data are available in this manuscript and its Supplementary Materials.

## Supplementary Materials

The following supporting information can be downloaded at https://www.mdpi.com/article/10.3390/jcm15166483/s1, Table S1: PRISMA Checklist; Table S2: Search Strategy.

## Abbreviations

**Abbreviation**	**Definition**
APR	Abdominoperineal resection
CI	Confidence interval
CRM	Circumferential resection margin
CRT	Chemoradiotherapy
GRADE	Grading of Recommendations Assessment, Development and Evaluation
IPSS	International Prostate Symptom Score
IIEF-5	International Index of Erectile Function-5
IPTW	Inverse Probability of Treatment Weighting
ISR	Intersphincteric resection
I^2^	Higgins inconsistency statistic
L-ISR	Laparoscopic intersphincteric resection
LARS	Low Anterior Resection Syndrome
MD	Mean difference
OR	Odds ratio
PRISMA	Preferred Reporting Items for Systematic Reviews and Meta-Analyses
PROSPERO	International Prospective Register of Systematic Reviews
PSM	Propensity score matching
RCT	Randomized controlled trial
R-ISR	Robotic intersphincteric resection
ROBINS-I	Risk Of Bias In Non-randomized Studies of Interventions
TME	Total mesorectal excision
FSFI	Female Sexual Function Index
EORTC	European Organization for Research and Treatment of Cancer
QLQ-C30	Quality of Life Questionnaire-Core 30
QLQ-CR29	Quality of Life Questionnaire-Colorectal Cancer Module 29
